# Teicoplanin-Modified HPLC Column as a Source of Experimental Parameters for Prediction of the Anticonvulsant Activity of 1,2,4-Triazole-3-Thiones by the Regression Models

**DOI:** 10.3390/ma13112650

**Published:** 2020-06-10

**Authors:** Jolanta Flieger, Anna Orzeł, Anna Kowalska-Kępczyńska, Magdalena Pizoń, Hanna Trębacz, Dariusz Majerek, Tomasz Plech, Wojciech Płaziński

**Affiliations:** 1 Department of Analytical Chemistry, Medical University of Lublin, Chodźki 4a, 20-093 Lublin, Poland; magdalena.pizon@umlub.pl; 2 Faculty of Medicine, Medical University of Lublin, Aleje Racławickie 1, 20-059 Lublin, Poland; anna.k.orzel@gmail.com; 3 Department of Biochemical Diagnostics, Medical University of Lublin, Staszica 16, 20-081 Lublin, Poland; anna.kowalska-kepczynska@umlub.pl; 4 Chair and Department of Biophysics, Medical University of Lublin, Jaczewskiego 4, 20-090 Lublin, Poland; hanna.trebacz@umlub.pl; 5 Department of Applied Mathematics, University of Technology, Nadbystrzycka 38D, 20-618 Lublin, Poland; majerek@gmail.com; 6 Department of Pharmacology, Faculty of Nursing and Health Sciences, Medical University of Lublin, Chodźki 4a, 20-093 Lublin, Poland; tomasz.plech@umlub.pl; 7 Jerzy Haber Institute of Catalysis and Surface Chemistry, Polish Academy of Sciences, Niezapominajek 8, 30-239 Krakow, Poland; wojtek_plazinski@o2.pl

**Keywords:** teicoplanin column, retention behavior, van’t Hoff relationship, phase ratio, phase transition, thermodynamic parameters, 1,2,4-triazole-3-thione derivatives, regression models, docking simulation, DFT results

## Abstract

The cell membrane is a complex system that consists of lipids, proteins, polysaccharides, and amphiphilic phospholipids. It plays an important role in ADME processes that are responsible for the final pharmaceutical effects of xenobiotics (bioavailability, activity). To study drug-membrane interaction at the molecular level, several high-performance liquid chromatography (HPLC) membrane model systems have been proposed which are mimicking mainly its lipid character. The aim of this work was to study interactions of new synthesized antiepileptic compounds of 4-alkyl-5-(3-chlorophenyl)-2,4-dihydro-3H-1,2,4-triazole-3-thione derivatives with Chirobiotic column containing glycoprotein ligand attached to the silica matrix. The affinity of the analytes to immobilized glycoprotein ligand was examined chromatographically in reversed-phase mode. The thermodynamics of interactions between bioactive compounds and teicoplanin was studied in terms of the van’t Hoff linear relationship ln k vs. 1/T in the range of 5–45 °C. Change in enthalpy (ΔH°), change in entropy (ΔS°) and change in Gibbs free energy (ΔG°) were estimated utilizing graphical extrapolation and interpolation methods. The density functional theory (DFT) approach and docking simulations were used to get the molecular interpretation and prove the obtained experimental results. Cross-correlations of chromatographic and thermodynamic parameters with non-empirical topological and quantum chemical indices suggest that the polarizability of analytes appears to be responsible for the interactions of the tested molecules with teicoplanin and, ultimately, their retention on the column. Experimental and theoretical parameters were subjected to statistical analysis using regression models. Partial least squares (PLS) regression model showed the usefulness of the experimentally measured parameter φ_0_ (MeOH) to discriminate between anticonvulsant active and inactive 1,2,4-triazole-3-thione derivatives. Obtained results point out the usefulness of interaction of potential anticonvulsants with glycoprotein class of compounds to anticipate their activity.

## 1. Introduction

**Scheme 1:**
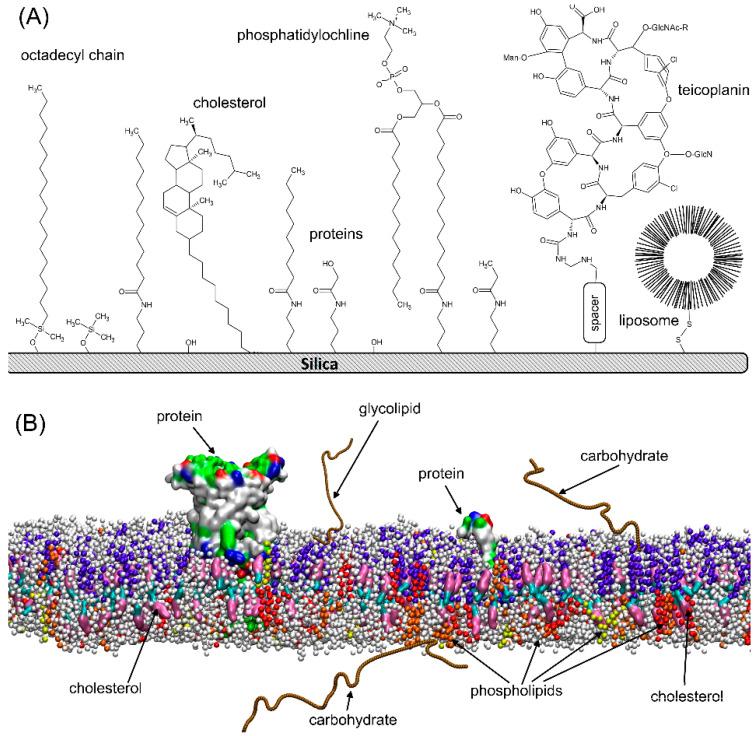
Schematic representations of stationary phases (**A**) simulating biological membrane (**B**). Surface-confined membranes at the top were obtained by covalently bonding phosphatidylcholine octadecylated silica (ODS), liposomes, cholesterol, proteins, and teicoplanin (the aim of current work) to porous silica.

In a living organism, xenobiotics undergo many biochemical and biophysical processes that are abbreviated as ADME, i.e., adsorption, distribution, metabolism, and elimination. Each of these stages depends in different ways on the lipophilicity of the xenobiotic. Therefore, many empirical and non-empirical lipophilicity scales are commonly used to predict the pharmacological activity of potential drugs. The process of penetration of exogenous substances through semipermeable membranes can be reflected by a partition coefficient. The oldest extra-thermodynamic extraction system, n-octanol-water, is considered to be the first model to simulate biological permeability. This two-phase system is a source of a parameter reflecting the relative lipophilic character of the compound, which is defined by log *P*, where *P* is the octanol-water partition coefficient [1,2]. However, in fact, the cell membrane is a complex system that consists of lipids, proteins and polysaccharides, and amphiphilic phospholipids. The drug’s ability to cross the membrane barrier is responsible for the observed range of pharmaceutical effects (bioavailability, activity). To study drug-membrane interaction at the molecular level, several membrane models have been created that mimic natural cell membranes, such as stationary phases in chromatography. Owing to this, it was possible to perform experiments under strictly defined and controlled conditions. Examples of surface-confined membranes are lipid vesicles, liposomes, Langmuir monolayers, and solid-supported lipid monolayers/bilayers (Scheme 1).

In the beginning, lipophilicity tests were performed by HPLC on octadecyl-dimethyl silane (ODS) phases with silica as solid support [3,4]. Chromatographic retention parameters (*k*, t_r_, log*k*, log*k*_w_) have been recognized as independent parameters of lipophilicity and are still successfully used to model biological lipophilicity [5,6,7,8]. In the 1990s, in order to model drug transport, solid phases known as artificial membranes (IAM) were synthesized [9,10,11,12,13]. Monolayers of phospholipid analogs were covalently bound to the silica surface or immobilized on conventional ODS columns. IAM-type stationary phases containing a phosphatidylcholine ligand or lipid-like, such as a cholesteric moiety covalently bonded to silica through a propylamine chain, are currently commercially available [14,15]. The retention mechanism on IAM columns involves significant intermolecular forces such as ionic bonds, electrostatic interactions between drug molecules, and the membrane, which is significant progress over historical octanol/water partition systems, or RP-HPLC exposing mainly hydrophobic interactions. That is why the relationships between the retention parameters (log k) obtained in the IAM column and log Po/w coefficients were rather weakly correlated [11,14,16].

Recently, immobilized liposome chromatography (ILC) raises great interest and hopes in simulating membrane-drug interactions. Many researchers emphasize its usefulness for the screening of drug partition in lipid bilayers [17,18,19,20,21]. The potential of this method is associated with the possibility of introducing additional ligands to the liposome surface. However, the widespread use of the ILC method limits the poor stability of liposomes and the need for self-preparation of the column.

To mimic the process of transport of xenobiotics across biological membranes, many solid carriers imitating biological membranes have been used to predict the interaction between the membrane and the drug, such as human skin, intestinal epithelium, and microvascular vascular endothelium [22,23,24].

The current work concerns the bioactivity prediction of newly synthesized substances with activity in the central nervous system (CNS). The therapeutic effect in CNS diseases is limited by a poor response to drug treatment. The main causes of therapeutic failure are mainly changes in drug uptake into target brain cells as a result of limited blood-brain barrier (BBB) ​​permeability, which impairs drug penetration through the brain [25,26,27]. Despite the fact that ordinary antiepileptic drugs (phenytoin, carbamazepine, oxcarbazepine, lamotrigine, phenobarbital, felbamate, valproic acid, topiramate) penetrate well into the brain, they have low efficiency. As a result, the therapy appears to be ineffective in approximately 30–40% of patients with epilepsy [25,28]. Therefore, reliable tests are required to predict whether newly synthesized compounds potentially exhibit anti-epileptic activity or only the ability to cross the blood-brain barrier (BBB). The most common experimental measures of BBB permeability are log BB (drug concentration in the brain divided by blood concentration) and log PS (product of the permeability surface). Both methods are time-consuming and expensive [29]. Other valuable alternatives to predicting BBB permeability can be provided by in silico calculation methods.

In this work, a Chirobiotic T column and a water-organic mobile phase operating in reversed-phase high-performance liquid chromatography (RP-HPLC) were used as a model to study a series of new candidates for anticonvulsants. The proposed RPLC is a dynamic two-phase system capable of mimicking biological membranes. Until now, various sorbents and artificial membranes have been used to simulate biological processes occurring at the interface. They partly reflected the structure of biological barriers, such as cholesterol in phosphatidylcholine columns, or focused only on demonstrating lipophilic properties, as in the case of classic ODS columns. The Chirobiotic T column was chosen for its structure representing the glycoprotein class of chemical compounds, anisotropic properties, reverse phase capability, and both types of chemical interaction, i.e., polar and hydrophobic, which makes it similar to complex and heterogeneous biological membranes.

Chirobiotic columns are obtained by attaching high purity macrocyclic glycoproteins to a spherical silica gel. The Chirobiotic T column has an attached amphoteric glycopeptide-eicoplanin. It has 23 chiral centers surrounding four cavities. Sites that can be both hydrogen bond donors and acceptors are located near seven aromatic rings. In 1994, Armstrong et al. described the first use of vancomycin, thiostreptone and rifamycin B macrocyclic antibiotic as chiral stationary phases (CSP) [30,31]. The chemical structure of these stationary phases enables various interactions with separated analytes, such as hydrophobic, π-π, dipole-dipole, hydrogen, electrostatic, ionic, and van der Waals interactions [32], as well as complex formation [33]. In turn, chiral recognition is possible due to a large number of stereogenic centers [34,35]. Teicoplanin contains amide linkages and sugar residues. It possesses an element of tertiary structure similar to proteins. In comparison to very large molecules like proteins, the teicoplanin-based stationary phase shows the dynamic mass transfer allowing separations for a wide range of compound classes.

The titled compounds belong to quite a new group of voltage-gated sodium channel blockers, the 4-alkyl-5-substituted-1,2,4-triazole-3-thione derivatives with a wide log *P* range (1.96–6.46). Docking simulations confirmed that these compounds bind to voltage-sensor domain IV (VSD4) located in mammalian NaV 1.7 proteins [36]. Importantly, the 1,24-triazole-3-thione derivatives turned out to possess strong anticonvulsant activity in various animal models of epilepsy, including MES and 6 Hz tests [37,38,39,40,41]. Simultaneously, they demonstrated many beneficial ADME-Tox properties, i.e., low neurotoxicity, lack of genotoxic effects observed in HepG2 cells, and the lack of negative impact on the viability of human cells when administered in concentrations necessary to achieve antiepileptic effects. Both pharmacological and physicochemical properties of the 4-alkyl-5-substituted-1,2,4-triazole-3-thione derivatives were highly affected by the structure of the alkyl substituents. The compounds containing unbranched chains produced stronger anticonvulsant effects than respective branched analogs. Their affinity towards respective molecular targets (i.e., VGSCc) was also stronger. However, the ability of the 1,2,4-triazole-based molecules to pass through the biological barriers (e.g., intestinal and blood-brain barriers) was much higher when the unbranched alkyl chain was replaced by the branched one [36,37,38,39,40,41]. Such a characteristic is of crucial importance in the development of new antiepileptics since the treatment of epilepsy is based mainly on oral medication. Therefore, the investigated 1,2,4-triazole derivatives (named TPR) seem to be an interesting group of antiepileptic drug candidates. In our previous papers [15,42], we examined these compounds on the cholesterol-based stationary phase, and the commercially available phosphatidylcholine biomimetic stationary phase [42]. Molecular dynamics simulations revealed the selective preferences of the phosphatidylcholine with respect to the compounds with either ether or sulfide moieties.

The aim of the current work concerns the retention mechanism of 1,2,4-triazole-3-thione derivatives on teicoplanin used as the stationary phase modifier. The thermodynamic van’t Hoff relationships and docking simulation were used for the above purpose. Retention behavior of studied molecules in this new dynamic two-phase system revealed some new factors influencing the observed chromatographic behavior as well as pharmacological activity. Furthermore, presented experimental (HPLC, DCS) and theoretical (docking simulation, DFT calculations, classification analysis) results are helpful for an understanding of the relationship between the structure vs. the properties of studied 1,2,4-triazole-3-thiones, and the function of teicoplanin as a new membrane mimicking the biological barrier.

## 2. Methods and Materials

### 2.1. Solutes and Reagents

The synthesis procedure of the derivatives of the 4-alkyl-5-substituted-1,2,4-triazole-3-thione derivatives (Table 1) was described previously [38]. Their structures were confirmed by gas chromatography-mass spectrometry (GC-MS), proton nuclear magnetic resonance (^1^H-NMR), carbon-13 nuclear magnetic resonance (^13^C-NMR), infrared spectroscopy (IR), and their purity was assessed by elemental analysis. The teicoplanin reference standard for DSC measurements was purchased from Sigma-Aldrich, Poznan, Poland). HPLC grade methanol (MeOH), and acetonitrile (ACN) were obtained from Merck (Darmstadt, Germany). Water purified by ULTRAPURE Millipore Direct-Q 3UV-R (Merck, Darmstadt, Germany) of the resistivity 18.2 MΩ cm was used to prepare all the aqueous solutions.

### 2.2. HPLC Measurement

The retention factors were measured with a liquid chromatograph, an Elite LaChrom HPLC Merck-Hitachi (Merck, Darmstadt, Germany) with DAD (Diode Array Detector L-2455) detector, pomp L-2130 and a manual sample injection valve equipped with a 20 µL loop and EZChrom Elite software (Merck) system manager as a data processor. The column was an Astec CHIROBIOTIC^TM^ (SUPELCO, Bellefonte, PA, USA); (150 mm × 4.6 mm I.D., 5 µm), pore size: 100Å. The injection of blank mobile phase volumes produced visible detector fluctuations that were used as the hold-up volume. The column was thermostated at 20 °C ± 0.1 using column thermostat Jetstream 2 Plus (100375, Knauer, Sigma-Aldrich, Poznan, Poland). The elution was carried out in the isocratic mode by the mobile phase consisting of methanol or acetonitrile in water. The mobile phase was filtered through a Nylon 66 membrane filter (0.45 μm) Whatman (Maidstone, England) by the use of a filtration apparatus.

The retention data were recorded at a flow-rate of 1 mL min^−1^ with online degassing using L-7612 solvent degasser at a wavelength chosen accordingly with the recorded spectra. The solutions (10^−4^ M to 10^−3^ M) were prepared by dissolving the solutes in the mobile phase. Typical injection volumes were 3 µL.

### 2.3. Differential Scanning Calorimetry (DSC) Experiments

Differential scanning calorimetry (DSC Q200 calorimeter, TA Instruments, Mettler-Toledo, Warsaw, Poland) was applied to study thermal transformations of the dry stationary phase. 5–8 mg samples were closed in aluminum pans (Tzero Pan) and temperature scanned from 0–210 °C at heating 5 °C/min in a nitrogen atmosphere. An empty pan was used as a reference.

### 2.4. The Methodology of Statistical Analysis

All calculations and visualizations were conducted in R environment [43] with a few packages which extend the possibilities of the base program, like caret [44], DALEX [45], ggpubr [46], random Forest [47], glmnet [48,49], and pls [50]. All linear regression analyses were performed using Excel (version from Office 97).

Two types of models were used to predict activity: classification and regression. For classification purposes, Regularized Logistic Regression, Random Forest, and Partial Least Squares Discriminant Analysis were used. In the regression approach, Random Forest, Partial Least Squares Regression, and Penalized Regression were applied to predict the dependent variable. The intuition standing behind all applied methods is to reduce the dimensionality and the collinearity in the pre-processing stage of analysis. To avoid estimation problems resulting from the high dimensionality of the predictive space and the lack of a sufficient number of observations, two methods of feature selection were used: Recursive Feature Elimination [51] and Simulated Annealing Feature Selection [52]. The recursive process and regularization were used to manage collinearity, while the high dimensionality was reduced using the partial least squares technique.

Data were randomly divided into train and test samples (they have 9 and 5 observations, respectively). The obtained models were verified on the test sample. During the model tuning procedure, a repeated 3-fold cross-validation method was used [53,54]. Models fit were assessed by Root Mean Square Error (RMSE), R^2^, and Mean Absolute Error (MAE), but the best fit was chosen based on RMSE, considering ED50 to predict response, while classification model’s accuracy was used to assess the model performance.

### 2.5. The Methodology of Molecular Modeling

The ligand molecules were prepared by using the Avogadro 1.1.1 software [55] and optimized within the UFF force field [56] (steepest descent algorithm). The flexible, optimized ligands were docked to the teicoplanin molecule according to the 1:1 binding stoichiometry. The teicoplanin structures found in the four following PDB entries were applied: 4mfl, 4pk0, 5awv, and 4pzj (X-ray resolutions varying from 0.16 to 0.23 nm). The AutoDock Vina software [57] was applied for docking simulations. The procedure of docking was carried out within the cuboid region covering the whole teicoplanin molecule. All the default procedures and algorithms implemented in AutoDock Vina were applied during docking simulations. The predicted binding energies were averaged over all the four teicoplanin structures. The more favorable binding mode is associated with the lower binding energy value; only the lowest energy value corresponding to the given ligand/teicoplanin system was accounted for during subsequent analyses. In accordance with the validated methodology of Vina-based docking, no explicit solvent was considered. However, the solvent effects are partially accounted for in an implicit manner by using the potentials calibrated against the data measured for solvent-containing systems.

The docking procedure was followed by the density functional theory (DFT)-based calculations. The geometry optimization was performed with the B3LYP nonlocal exchange correlation functional [58] and the 6-311 + G basis set [59]. The presence of the implicit solvent (methanol) was accounted for by applying the COSMO approach. The possibility of introducing the explicit solvent is limited due to both computational efficiency reasons and the large configurational complexity of the solvent-containing systems. The default convergence criteria implemented in Gaussian09 [60] were accepted. Calculations involved the teicoplanin molecule, molecules of all ligands, and the ligand-teicoplanin complexes. The initial structures were taken from the docking results; wherever necessary, the part of the system was removed to obtain the derided composition. The binding energies were calculated from the energy difference between (i) energy of the complex; (ii) the sum of the energies of isolated teicoplanin and ligand molecules.

### 2.6. Evaluation of Anticonvulsant Activity

The details of the anticonvulsant activity evaluation procedure and subsequent results are partially described in our previous works [15,37,42]. The anticonvulsant activity of the investigated compounds was measured in mice maximal electroshock (MES)-induced seizure model of epilepsy. The MES model is considered to mimic generalized tonic-clonic seizures in humans. The experiments were conducted on adult Swiss albino mice (males) weighing 20–25 g, which were kept in colony cages with free access to food and tap water. After one week of acclimatization, the animals were randomly divided into groups consisting of 8 mice. The investigated compounds were suspended in a 1% solution of Tween 80 in distilled water and administered intraperitoneally in a volume of 5 mL/kg. Electroconvulsions were produced by an alternating current (0.2 s stimulus duration; 500 V, 50 Hz, fixed current intensity of 25 mA) delivered via ear-clip electrodes by a Type 221 Rodent Shocker generator (Hugo Sachs Elektronik, Freiburg, Germany). The abolition of a hind-limb tonic extension (HLTE) was taken as a criterion of the anticonvulsant activity. The tested compounds were administered in increasing doses in order to calculate ED50 values using the log-probit method by Litchfield and Wilcoxon. Each ED50 reflects the dose of a compound required to protect 50% of the animals tested against maximal electroshock (MES)-induced seizures.

## 3. Results and Discussion

Two classical models usually used to interpret the dependence of the retention factor (*k*) on the mobile phase composition and the temperature are described by the Soczewiński-Wachtmeister equation (Section 3.1) known as the linear solvent strength model (LSSM), and the second one concerning thermodynamic studies represented by the Van’t Hoff plot (Section 3.2).

### 3.1. Retention Behavior on Teicoplanin Column

**Figure 1:**
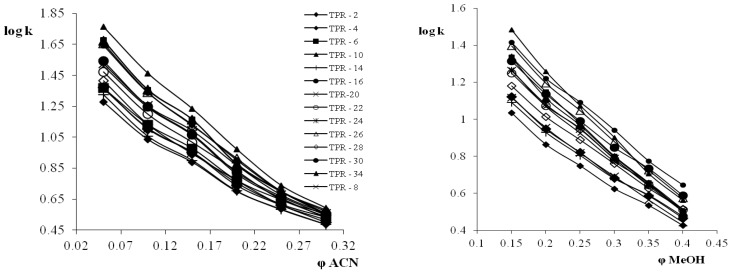
Plots of log k against φ (*v*/*v*) methanol (MeOH), acetonitrile (ACN) in the mobile phase obtained on CHIROBIOTIC^TM^ column.

The main purpose of the initial experiments was to examine the retention behavior of new 1,2,4-triazole-3-thion derivatives on the teicoplanin column. Teicoplanin stationary phase may be used under reversed-phase (RP) conditions with low proportions of polar organic solvent to an aqueous component. Retention and selectivity on the teicoplanin column depend primarily on the concentration and nature of the organic modifier (methanol or acetonitrile), similarly to the most reversed-phase systems. Higher concentrations of the organic modifier resulted in lower retention, typically for reversed-phase (RP) systems (Figure 1).

The retention behavior of the analyzed substances was tested in polycratic conditions. Methanol concentration was ranging from 0.15 to 0.40 of the volume fraction of the organic modifier in the eluent (φ), whereas acetonitrile concentration was from 0.05 to 0.3 φ.

Obtained linear dependences of log k on φ, are described by the Soczewiński-Wachtmeister Equation [61]:

log *k* = − *S*φ + log *k*_w_ (1)

Based on these relationships, log *k* values extrapolated to 100% water (log *k*_w_) and the *S* values expressing the slope of the following equation were determined. The chromatographic lipophilicity parameters, for two selected eluent systems (A and B), are presented in Table 2. The coefficients of determination (R^2^) of the above correlations were higher than 0.9 for the acetonitrile-water mobile phase (A) as well as in the case of methanol as an eluent modifier (B). The high values of F and small standard correlation errors (s_e_ < 0.05) also testify to the perfect matching of the dependencies to the straight line.

The obtained retention parameters appear to favor the acetonitrile-type mobile phase. The average slope values of the relationship log *k* vs. φ are 25% higher for acetonitrile- (*S* = −3.816) containing eluent system in comparison to methanol (*S* = −2.846).

The log *k*_w_ parameter is the theoretical retention of the analyte in pure water or buffer. It is very often used in quantitative structure–activity relationships (QSAR) as a lipophilicity parameter. Theoretically, this parameter eliminates the influence of the organic modifier, however, it should be remembered that it refers rather to interphase, which is the stationary phase changed by the presence of mobile phase molecules.

In 1993, Valko and Sleger [62] introduced another parameter that is used to compare lipophilicity, i.e., φ_0_. This parameter illustrates the composition of the eluent for which *k* is 1, i.e., the amount of substance in the mobile and stationary phases equals each other. The φ values are determined by the interpolation method of the relationship log *k* = f(φ). It turns out that this parameter is less dependent on the type of organic modifier used in the eluent, and it is better reproducible and repeatable compared to log *k*_w_ [63,64].

The log *k*_w_ vs. *S* correlation graphs are used to study the structural similarity of analytes. For related substances that do not differ in donor-acceptor properties, statistically, significant relationship *S* vs. log *k*_w_ should be observed. For both elution systems (methanol-water, acetonitrile-water) satisfactory correlations described by the following linear regression equations were obtained:

MeOH/Water: log *k*_w_ = −0.4205(±0.03939)*S* + 0.4540(±0.1139) n = 14, R^2^ = 0.8976, F = 113.9818, s_e_ = 0.062873 (2)

ACN/Water: log *k*_w_ = −0.3359(0.009338)*S* + 0.3145(0.36408) n = 14, R^2^ = 0.9901, F = 1294.178, s_e_ = 0.019135 (3)

where numbers in parentheses correspond to 95% confidence limits; *n* is the number of the compounds; *R*^2^ is the squared correlation coefficient; s_e_ is the standard deviation and F is the Fisher’s test.

The obtained results prove the usefulness of the Chirobiotic T column for the assessment of structural similarities of the investigated 1,2,4-triazole-3-thiones. Furthermore, significant correlations, presented above, additionally imply that the retention of the investigated analytes is controlled by the same intermolecular forces.

**Figure 2:**
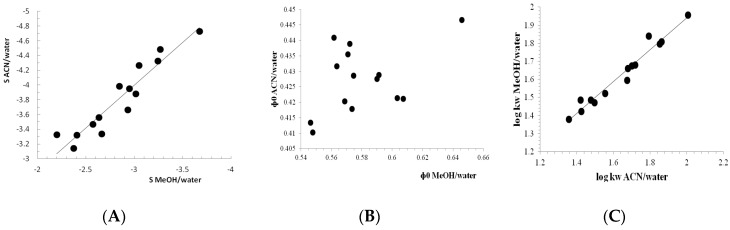
The cross relationship between the chromatographic lipophilicity parameters: *S* (**A**), φ_0_ (**B**), and log *k*_w_ (**C**), measured on chirobiotic column by the use of different eluent systems: ACN/water and MeOH/water.

Regardless of the type of organic modifier in the mobile phase, the log *k*_w_ values and the *S* parameters are very similar to each other. These similarities are reflected by the relationship of log *k*_w_ (MeOH/Water) vs. log *k*_w_ (ACN/Water), and *S* (MeOH/Water) vs. *S* (ACN/Water), (Figure 2A,C) which has a slope close to one, a low value of the intercept, close to one correlation coefficient, and a small standard error. Only the φ_0_ values are not correlated with each other in the tested elution systems.

log *k*_w_ (MeOH/Water) = 0.9952(±0.06705) log *k*_w_ (ACN/Water) + 0.0541(±0.1087) (4)

*n* = 14, R^2^=0.9443; s_e_ = 0.04638; F = 220.3161

S (MeOH/Water) = 0.7682(±0.0777) S (ACN/Water) + 0.08605(±0.2988)(5)

*n* = 14, R^2^ = 0.8907; s_e_ = 0.1384; F = 97.8039

φ_0_ (MeOH/Water) = 1.1149(±0.6355) φ_0_ (ACN/Water) − 0.1033(±0.2716) (6)

*n* = 14, R^2^ = 0.2041; s_e_ = 0.02442; F = 3.0779

This result is related to the linearity of the Soczewiński-Wachtmeister relationship. Linear relationships are obtained in limited regions of the organic modifier concentration. Usually, deviations from linearity occur at higher modifier concentrations. This effect is due to excessive adsorption of the organic solvent in the stationary phase. The concept of the adsorbed layer was first suggested by Knox and Pryde [65] and confirmed by Kazakevich et al. [66]. As with other RP systems, the retention process on the teicoplanin column with elution systems containing methanol/acetonitrile can also be modeled using the Soczewiński-Wachtmeister relationship. However, the obtained relationships were better correlated in the case of methanol playing the role of solvent (with a correlation coefficient approaching 0.99 in the entire range of tested concentrations). Thus, in this case, the results of extrapolation and interpolation cannot be questioned.

In turn, correlations between log *k* and acetonitrile content were slightly worse with an average R^2^ value approaching 0.98, suggesting that acetonitrile may form a multilayer which is adsorbed on top of the bound phase. In this case, the Soczewiński-Wachtmeister relationship may not be true in a wide range of organic content. Unlike methanol, excessive adsorption of acetonitrile at the surface can cause a reduction in analyte-surface interaction and, consequently, the corresponding retention. This is the main reason for the disturbances from the linear shape of the Soczewiński-Wachtmeister graphs appearing in the form of a concave shape. Since deviations from linearity usually occur at higher acetonitrile concentrations, the most disturbing is the interpolation results obtained in acetonitrile (φ_0_).

The lipophilicity parameters can also be determined through quantum-chemical calculations. The theoretical method comprised calculations of AC log *P* using the in silico model, based on Hansch and Leo’s approach [67], from the VCCLAB online calculator [68].

**Figure 3:**
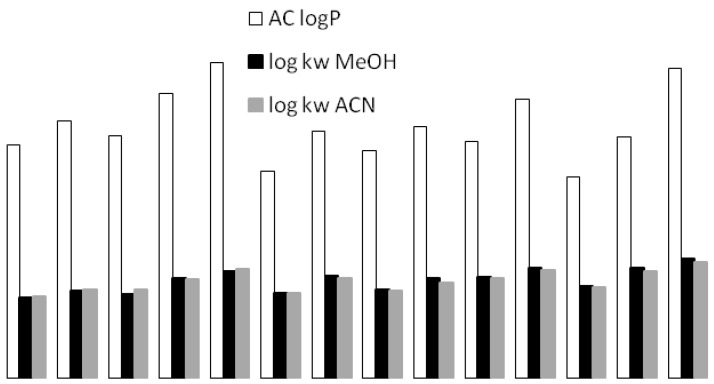
Comparison of different lipophilicity scales: chromatographic (log *k*_w_ MeOH, log *k*_w_ ACN, and calculated theoretically (AC log *P*). Values were taken from the experimental database VCCLAB (ALIGPS 2.1).

As can be seen in Figure 3, the theoretical hydrophobicity values expressed by AC log *P* are much higher than the chromatographic scale of lipophilicity represented by log *k*_w_ values, either in the methanol or acetonitrile eluent. Thus, in a two-phase system in an aqueous-organic environment, the tested compounds showed more uniform hydrophobicity, being at least two times lower in comparison to the calculated values. This may indicate the induction of the dipole moment of the studied molecules by the polar environment, which results in the weakening of hydrophobic interactions.

### 3.2. Thermodynamic Studies

Temperature effects also provide very useful information to explain retention mechanisms and better understand analyte behavior in the chromatographic systems. Moreover, elevated temperatures offer many advantages in liquid chromatography. An increase in temperature can improve the sample throughput because van Deemter minima are transferred to higher flow rates. Besides, as the temperature rises, the water behaves more like an organic solvent, therefore, it is possible to reduce the amount of organic modifier at elevated temperatures. The thermodynamic parameters were obtained based on the temperature dependence of the retention factor (*k*) known as the van‘t Hoff relationship:

ln *k* = −ΔH°/RT + ΔS°/R + ln Φ (7)

where Φ is the phase ratio; (V_s_/V_M_) is the ratio of the stationary phase volume to the mobile phase volume, the standard enthalpy change (ΔH°), and the standard entropy change (ΔS°); R is the universal gas constant (8.31 J mol^−1^ K^−1^); T is the absolute temperature and *k* is the retention factor in chromatography.

**Figure 4:**
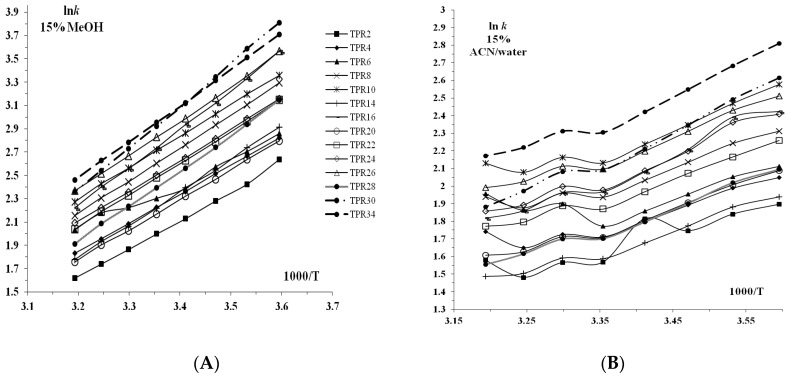
Van’t Hoff plots between the natural logarithms of the retention factors vs. 1000/T for the examined compounds. (**A**) 15% methanol as the mobile phase; (**B**) 15% acetonitrile as the mobile phase.

The individual van‘t Hoff plots representing log *k* vs. 1/T were generated under isocratic conditions involving the following elution systems: 15% ACN and 15% MeOH (Figure 4A,B). The obtained graphs were almost linear with the sufficient values of the determination coefficient (R^2^ > 0.9) only for methanol-containing eluent systems. Sufficient linearity of the van’t Hoff graphs proves that the retention mechanism is constant over the temperature range under consideration, and makes them useful in measuring thermodynamic parameters such as the ΔH°, ΔS°, and free energy of transfer (ΔG°) of 1,2,4-triazoles from mobile phases to the investigated sorbent. In the further static analysis, only parameters obtained in the methanol-water system were taken into account as the most reliable. From ln *k* vs. 1/T plots, the slope and intercept are −ΔH°/R and ΔS°/R + ln Φ, respectively. The Gibbs free energy change (ΔG°) was calculated by the Gibbs-Helmholtz equation:

ΔG° = ΔH° − TΔS°(8)

In contrast to methanol, acetonitrile-containing mobile phases formed non-linear van’t Hoff plots. According to Chester and Coym’s study [69], the disturbances in a van’t Hoff plot are the consequences of a phase transition in the stationary phase or changes in the phase ratio. In this case, the thermodynamic parameters derived from the van’t Hoff plots cannot be trusted. Determination of the phase ration (Φ) in chromatography is the subject of debate. There are some methods of its determination proposed among others by Dorsey [70,71]. However, most thermodynamic studies assume that the phase ratio values are constant. Although, there is some evidence that the volume of the stationary phase, which is accessible to analyzed molecules, changes with the mobile phase composition and temperature. There is evidence, especially for the ODS bonded column, of structure collapse changing its conformation, in the case of high water content in the eluent system [66] or temperature increase [72]. Moreover, there is evidence that the phase ratio is also solute-dependent [69]. As interphase changes its conformation and finally, its accessible volume with temperature, it will further affect interactions with different analytes by changing steric conditions and efficiency of solute diffusion into the stationary phase. We also cannot ignore the accessibility of the mobile phase to solutes, because some of them are permanently excluded from the liquid phase owing to unfavorable charges.

The impact of various factors, i.e., phase transition, type of analyte, and especially their charges, temperature change and, even rarely considered in HPLC, pressure changes that affect the volume of sorbent available for interaction through the compression effect, on thermodynamic parameters including the enthalpy value, is very complex and not completely clear. In addition, according to Chester and Coym [69], these factors often offset each other. Efforts to obtain reliable values for the corresponding parameters are only relevant when we compare thermodynamic parameters measured on different columns. As in our work only one stationary phase was used, no phase transition for teicoplanin was observed in the studied temperature range (Section 3.3), so then the thermodynamic parameters were calculated directly from the van’t Hoff curves if they were sufficiently linear. For this reason, the further static analysis included only the parameters obtained in the methanol-water system as the most reliable.

All parameters calculated based on the simple linear regression analysis have been collected in Table 3. The obtained ΔS° values (ΔS°*) are biased by the constant error, namely R ln Φ which could be completely canceled for ΔΔS calculations.

The obtained values of the enthalpy change were all negative, but the more negative ΔH° values were obtained for methanol-containing eluent systems in comparison to acetonitrile ones, indicating the existence of more favorable, exothermic interactions between the 1,2,4-triazole derivatives and the teicoplanin stationary phase. The changes in enthalpy are 1000 times higher as compared to changes in entropy. Therefore, the free energy is favorable (ΔG° < 0), mainly due to the enthalpic contribution for both eluents.

The ΔH° values for a series of the compounds containing unbranched chains TPR 14, 16, 28, 30 became the most negative (exothermic). It was proved previously that these compounds possess stronger anticonvulsant effects, the highest affinity towards respective molecular targets, and the smaller ability to pass through the biological barriers than respective branched analogs [37]. The obtained chromatographic results show that the incorporation of an unbranched, long hydrocarbon chain into the entire structure significantly enhanced interactions with the model membrane. The ΔH° values for increasingly hydrophobic analytes containing more carbon atoms should become more negative leading to larger values of *k*. This effect is shown in Figure 1. The more hydrophobic analytes containing 7 carbon atoms (TPR 34) in substituent have larger retention than those with shorter chain lengths containing 4 carbon atoms (TPR 2, TPR 20). To study the interactions’ pattern between examined compounds and teicoplanin, docking simulations have been performed.

### 3.3. The Phase Transition of Teicoplanin

**Figure 5:**
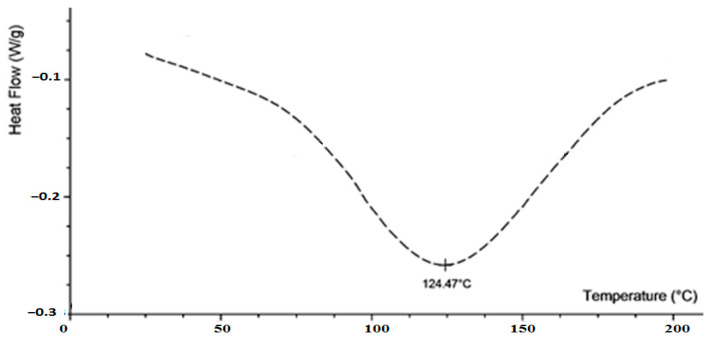
Phase transitions for the dry stationary phase checked by DSC (differential scanning calorimetry).

To examine a phase transition, differential scanning calorimetry (DSC) was applied. Obtained results showed, over a temperature range of 30–150 °C, that the melting temperature of neat teicoplanin equals 124.47 °C (Figure 5). Dry teicoplanin did not undergo any phase transition under studied conditions in the range from 5 to 40 °C. As it is above the examined range, it indicates that rather the phase ratio or structure of the individual solutes accessible for the different stationary phase volume cause observed non-linearities in the van’t Hoff plots.

### 3.4. Selectivity of Teicpolanin Column towards Studied Triazoles

Chester and Coym elaborated a procedure [69] useful for estimation of transfer enthalpy which is completely independent of phase ratio. The molecular difference method enables to measure transfer enthalpy for the molecular difference between two solutes. The method can be applied for similar molecules possessing access to the same stationary phase volume. In this case, we will be able to assume that they encounter the same thermodynamic phase ratio. Chester and Coym’s method is valid at constant pressure at which compression effects of the stationary phase cannot be observed. The slope of the relationship between ln *α* versus 1/T equals:

(9) $$slope=\left(\frac{\partial \ln\alpha }{\partial (1/T)}\right)=\frac{-\Delta H_{d}{}^{0}}{R}$$

where ΔH°d is the partial molar enthalpy of transfer of the molecular difference for each of two selected series members; α is the quotient of the retention coefficients (*k*) for selected pairs of compounds showing structural differences (α = *k*_2_/*k*_1_, where *k*_2_ > *k*_1_).

**Figure 6:**
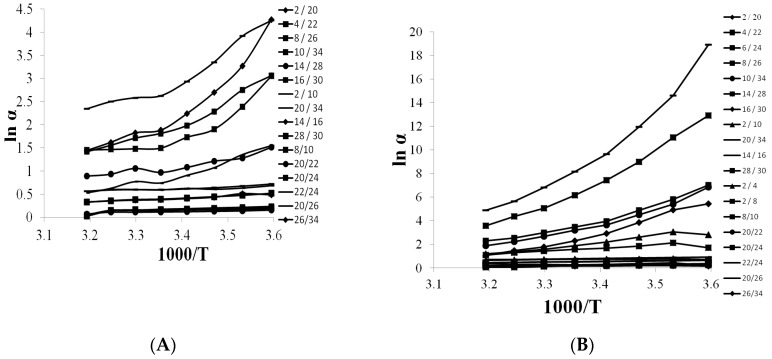
The selectivity plot for distinguishing pairs that differ with methylene or methyl moieties, heteroatoms, or isomeric form examined on Chirobiotic column by the use of 15% ACN (**A**) and 15% MeOH (**B**).

**Figure 7:**
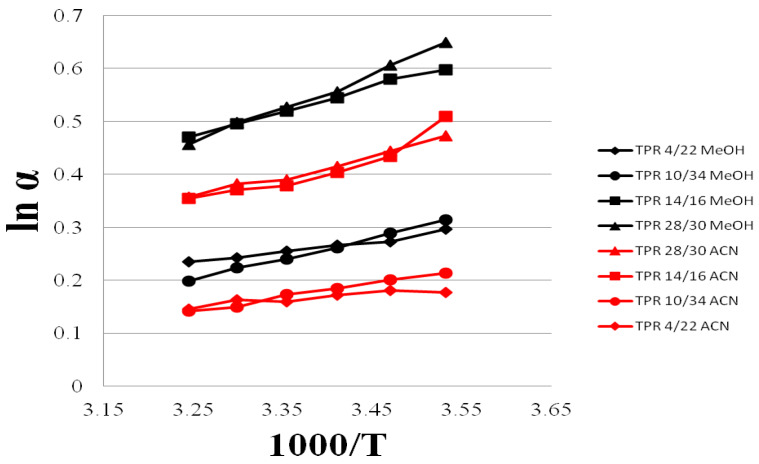
The phase ratio-independent selectivity curves for analytes differing with the heteroatoms at R1 substituents (TPR 14/16 and 28/30) and the methylene group (TPR 4/22 and TPR 10/34) obtained on Chirobiotic column. Experimental conditions: teicoplanin column (CHIROBIOTIC^TM^), 15% ACN (red lines) and 15% MeOH (black lines) mobile phases, at a flow rate of 1 mL/min.

For most distinguished pairs, some degree of the phase-ratio dependence causes the not linear shape of ln *α* = f(1/T) relationships (Figure 6). It suggests that they access to different stationary phase volume. Therefore, we selected only the linear dependencies of ln *α* versus 1/T for both eluent systems studied, characterized by the determination coefficients higher than 0.97 (Figure 7).

Figure 7 presents the relationship between ln *α* for analytes differing with the heteroatoms at R1 substituents (TPR 14/16 and 28/30) in the imidazole ring and selectivity between TPR 4/22 and TPR 10/34 (since the molecular difference between them is a methylene group), against the inverse temperature (1/T). When analyzing Figure 6, it can be concluded that chosen pairs access the same stationary phase volume. This suggests also that the transfer enthalpy for heteroatoms: sulfur and oxygen being their molecular difference, differs from each other. Since the selectivity plot is not affected by the phase ratio, the presence of the heteroatoms is responsible for access of these compounds to the stationary phase.

The transfer enthalpy from the mobile phase containing methanol to the teicoplanin stationary phase for the heteroatoms calculated from the slope of ln *α* against 1/T is about −3.77 kJ/mol for series without methylene linker and −5.46 kJ/mol for series containing methylene linker. For pairs differing in methylene linker: TPR 4/22 and TPR 10/34, enthalpy of transfer varies from −1.71 to −3.28 kJ/mol. Corresponding values of the transfer enthalpy from mobile phase containing ACN are 30–40% less negative, and they are expressed by the following for heteroatom selectivity: −2.80 kJ/mol (TPR14/16), −3.29 kJ/mol (TPR 28/30), and for methylene selectivity: −0.93 kJ/mol (TPR 4/22), −2.26 kJ/mol (TPR 10/34).

### 3.5. Cross-Correlation of Chromatographic and Thermodynamic Parameters with Theoretical Structural Descriptors

In the aim to find a descriptor which is responsible for the interactions of the tested molecules with teicoplanin and ultimately contributes their retention on the column, cross-correlations of chromatographic and thermodynamic parameters with non-empirical topological and quantum chemical indices have been performed. In Table 4, cross-correlation results represented by the determination coefficient (R^2^) have been collected for the relationship between experimental retention parameter log k_w_ and the Gibbs free energy change ΔG° estimated in each eluent system studied and theoretical structural descriptors, such as topological and quantum chemical indicators.

The statistically significant correlation was obtained for polarizability values and ΔG° estimated for the methanol-containing mobile phase. It could be concluded that the strength of the interactions between teicoplanin and examined molecules is related to their polarizability, in that case. It means that the induced dipole moment of examined molecules formed in the methanol/water solvent system is associated with an appropriate and proportional strength of interactions with teicoplanin. This conclusion could be explained by solvent polarity expressed by the empirical E^N^_T_ scale. In this scale, methanol polarity is 0.762 whereas acetonitrile is 0.046. The polarity of water was assumed to be 1. So, methanol in water will have higher solvation properties in comparison to acetonitrile/water.

These correlations are in line with previous suggestions regarding dipole moment induction by the polar environment. What is more, the theoretically calculated polarization value seems to be a good tool in predicting the retention properties of tested molecules on a teicoplanin column with a methanol/water solvent, and thus, to assess the thermodynamics of absorption of these new therapeutic substances. It should also be noted that the low correlation with the BBB parameter representing the possibility of crossing the blood-brain barrier confirms the observation of other researchers that the passage of BBB is not always associated with anticonvulsant activity. To examine the mechanism of interaction between teicoplanin and the derivatives tested, a docking simulation was performed.

### 3.6. Study of Interactions of Teicoplanin with Investigated Triazoles by Docking Simulation

The density functional theory (DFT) approach and docking simulations were used to get the molecular interpretation and prove the obtained experimental results. The binding energies found during docking simulations and DFT calculations are given in Table 5 and graphically illustrated in Figure 7. In general, the obtained values exhibit very similar magnitude and vary from each other by less than 1 kJ/mol. The latter value is well below the accuracy threshold characteristic of the majority of standard molecular modeling methods, including those applied in our study. Thus, we refrain from a detailed, quantitative analysis of the data collected in Table 5. However, it is worth noting that both the magnitude of the calculated binding energies as well as their relatively minor scatter are in line with the corresponding experimental data (see Table 3 and Figure 3). Let us mention that the docking procedure included the whole teicoplanin molecule and resulted in nearly uniform binding modes, depending solely on the molecular topology of the studied ligands (see discussion below); this additionally indicates the meaningfulness of the obtained results.

**Figure 8:**
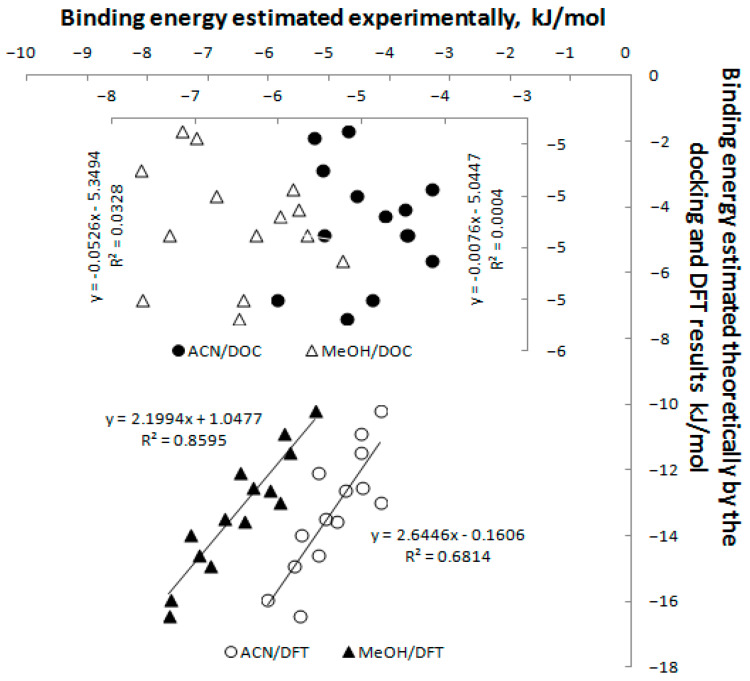
The comparison of the Gibbs free energy change (ΔG°) estimated experimentally on Chirobiotic column with methanol (MeOH)- or acetonitrile (ACN)-containing eluent system and obtained by the use of docking simulation (insert) and DFT calculations.

The energies extracted from the DFT calculations exhibit a much higher correlation with experimentally-determined energy values (see Figure 8). However, the determined values are systematically higher, which can be ascribed to the lack of both the explicit solvent and any additional protocol to compensate the effects following this approximation. Such compensating approaches are inherent to the Vina-based docking, described above, which is probably the main reason for the discrepancies between DFT and docking-based energies.

**Figure 9:**
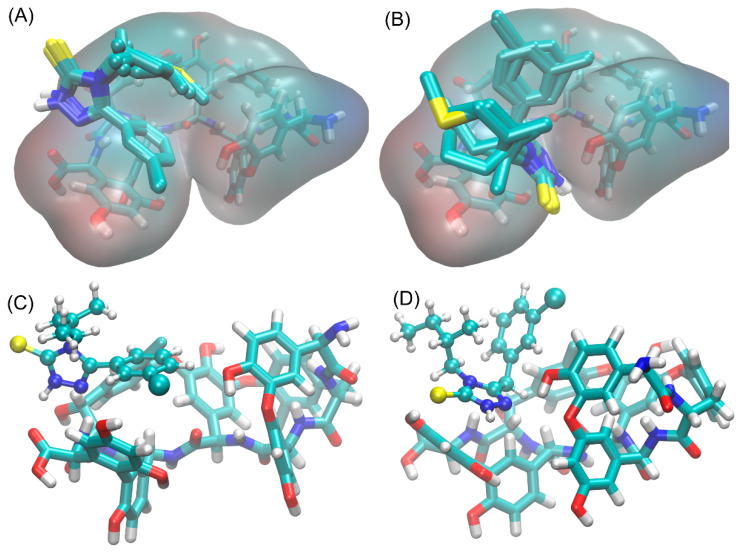
(**A**,**B**) Graphical illustration of the docking results. The two groups of ligands are considered separately, i.e., those that either do (**A**) or do not (**B**) contain a methylene group between triazole and phenyl rings. The pictures show the identified locations of the ligand molecules (stick representation) interacting with teicoplanin (stick plus surface representation). (**C**,**D**) Graphical illustration of the DFT-optimized structures of complexes, shown on the example of the TRP-10 (**C**) and TRP-26 (**D**) compounds. The ligands are shown in the ball-and-stick representation.

The results of both the docking and DFT studies have been analyzed with respect to the mechanistic interaction pattern that may be significant in the context of the interpretation of the obtained binding energy values. The graphical illustration of the docking results is given in Figure 9A,B whereas the exemplary visualization of the DFT-based geometry optimization is given in Figure 9C,D. In general, the two distinct binding patterns were identified during the docking procedure which were maintained after subsequent DFT calculations. The first of them is exhibited by compounds with direct (i.e., via single covalent bond) linkage between triazole and phenyl rings (TRP-2-TRP-16) whereas the second, by compounds that contain the additional methylene moiety between those two rings (TRP-20-TRP-34). Within each of these groups, the binding mode is very similar and the main differences concern only the region of the triazole ring substituent (−R in Figure 9), the type of which differs from one compound to another. These small structural divergences may be responsible for the resulting minor differences in binding energies. Moreover, the mentioned ring substituent does not exhibit any direct contact with the teicoplanin molecule which, additionally, can be associated with its limited influence on the total binding energy. Finally, the –R substituents are usually flexible, which implies a high entropic component of the associated binding free energies; this factor is hardly reproduced by the static docking simulations and DFT calculations.

As identified by the docking study, the binding pattern exhibited by the first group of compounds is characterized by the close contact of the ligand chlorophenyl group with the dihydroxy phenyl moiety of teicoplanin. The parallel arrangement of these two rings indicates the presence of π-π interactions. At the same time, the triazole ring is rotated by about 60 deg with respect to the phenyl ring and maintains close contact with aliphatic and aromatic hydrogen atoms that belong to the hydroxyl methylene and chlorophenyl groups of teicoplanin, respectively. The arrangement of those contacts speaks for the presence of the attractive H-π stacking interactions. The remaining part of the ligand molecule (i.e., the −R substituent) does not exhibit any close contacts with teicoplanin but rather tends to create intramolecular interactions with the chlorophenyl group.

Most of the above-mentioned patterns are maintained after DFT-based geometry optimization. The most important differences include: (i) The disruption of the initially parallel arrangement of the ligand chlorophenyl group with respect to the dihydroxy phenyl moiety of teicoplanin. The twisted alignment speaks for the contribution of both π-π interactions and H-π stacking; (ii) creating the hydrogen bonding between the triazole ring and the carboxyl group of teicoplanin. At the same time, the −R substituent is moved even further from the teicoplanin molecule but this type of interaction does not seem to be relevant for the main binding driving force.

Ligands in the second group contain additional methylene moiety between two ring structures in their molecule. This has significant implications for the binding pattern in comparison to the first group, discussed above. Namely, now the triazole ring interacts by the π-π interactions with the chlorophenyl group of teicoplanin. The attractiveness of this interaction pattern is additionally enhanced by the proximity of the hydroxyl group of the teicoplanin with electronegative nitrogen atoms that create the triazole ring. The second ring moiety creates the H-π stacking interactions that involve aliphatic and aromatic hydrogen atoms belonging to the hydroxy methylene and chlorophenyl groups of teicoplanin, respectively. Again, the –R substituent does not maintain any close contacts with teicoplanin molecule but, in the case of larger substituents, may interact with the chlorophenyl group of its own molecule.

The DFT calculations reveal a series of slight changes in the described above patterns of interactions. Namely, the triazole ring arrangement with respect to the chlorophenyl group of teicoplanin is twisted in relation to the initially parallel arrangement. This speaks for the larger contribution of the H-π stacking interactions involving those two groups. Secondly, the hydroxyphenyl group of the teicoplanin forms a fully developed hydrogen bond with the triazole ring. At the same time, the −R substituent and the group responsible for the H-π stacking interactions identified during docking simulations are moved further away from the teicoplanin molecule, which results in reducing the magnitude of the corresponding interactions.

Summarizing, the interactions of the studied compounds with teicoplanin exhibit well-defined structural patterns, common for all compounds following the same molecular topology. The main driving force for binding seems to be the π-π and H-π interactions, supported by hydrogen bonding which involves the triazole ring. The limited contacts between the −R substituent and teicoplanin explain the small scatter of binding free energies determined across the studied group of compounds.

As can be seen in Table 3; Table 5, Gibbs free energy values obtained by docking simulation differ from those obtained experimentally in a chromatographic system. The docking-derived binding energies exhibit a very weak correlation with those obtained experimentally, however, some analogy between them can be noticed (Figure 8). The ΔΔG° values representing teicoplanin selectivity in relation to the heteroatom type (oxygen, sulfur), i.e., ΔΔG° TPR 14/16 and ΔΔG° TPR 28/30 are 0.23 and 0.25 kJ/mol in docking, 1.34 and 1.37 kJ/mol in the methanol system, and in the system with acetonitrile, 1.02 and 1.03 kJ/mol. As for the selectivity towards the methylene group, the values obtained by docking simulation for pairs TPR 4/22 and TPR 10/34 are 0.35 and 0.62 kJ/mol, respectively, while obtained in the methanol system is 0.65 kJ/mol for both pairs of compounds, and in the system with acetonitrile is 0.4 kJ/mol. The ΔΔG° values obtained experimentally are a little higher than those obtained by the docking method, which once again confirms the contribution of the solvent involved in the interaction process.

In contrast, correlations of the DFT results versus chromatographically-obtained ΔG° in both eluent systems are surprisingly consistent. The R^2^ values are the following: 0.8595 for MeOH- containing eluent and a little bit worse for ACN, namely 0.6814 (Figure 8).

### 3.7. Statistical Analysis of Thermodynamic and Chromatographic Parameters for Activity Evaluation

All collected data on chromatographic retention, thermodynamic parameters, and anticonvulsant activity expressed as IC50 were subjected to statistical analysis to find the method and parameter most useful for screening candidates for anticonvulsants.

#### 3.7.1. Activity Division

**Figure 10:**
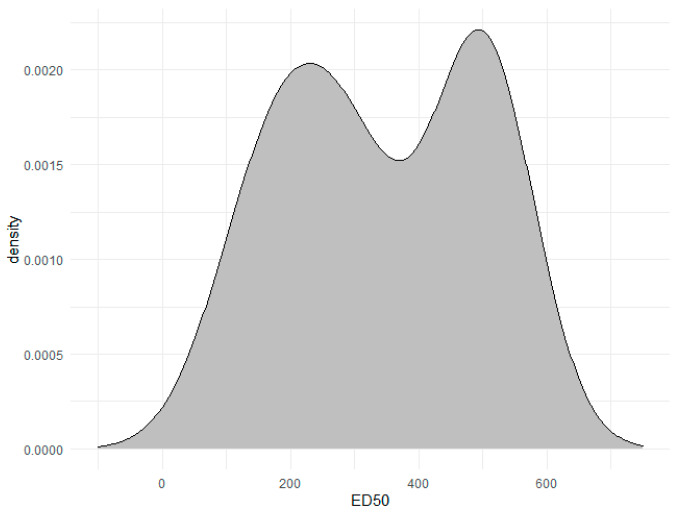
The distribution of activity parameter.

Figure 10 illustrates the distribution of the activity parameter. The clear bi-modal distribution suggests that there is a mixture of two populations of observation, one with the lower activity described by higher ED50 parameter, and the second of higher activity individuals with lower ED50. Based on the distribution, the optimal discrimination boundary for ED50 is 350.

#### 3.7.2. Comparison of Classification Models

During Regularized Logistic Regression (PLR) model tuning process, best accuracy (0.83) was obtained for λ = 0.1 and α ≈ 0.016 on the training dataset. Validation procedure conducted on test samples shows 0.8 model prediction, so quite similar to the accuracy of train samples. Therefore, we can say our model is not overfitted (Table 6).

Two methods of feature selection (Recursive Feature Elimination (RFE) and Simulated Annealing (SA) Feature Selection) were applied to extract the best set of predictors. Considering both selection methods, the following set of predictors was selected as optimal: RFE: (MeOH), S (MeOH), (ACN), log k (25% ACN); and SA: G (15% MeOH), log k (20%MeOH), log k (10% ACN), log k (15% ACN), log k 25% ACN), log kw (MeOH). Better precision of the RF model on train samples was obtained for the RFE algorithm of the initial selection of variables. So, the final RF model was built with RFE selection.

**Figure 11:**
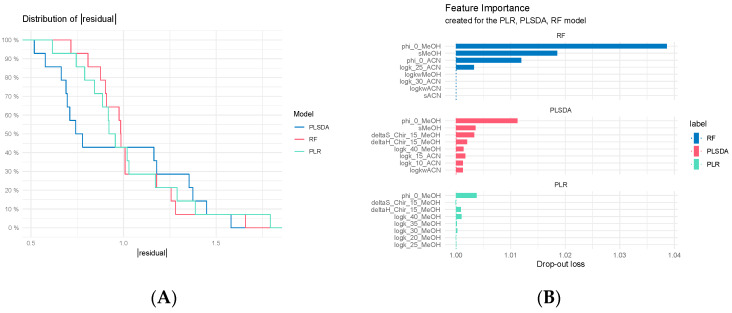
Distribution of residuals (**A**) and variable importance for the set of optimal RFE (Recursive Feature Elimination) predictors (**B**) of three estimated models.

Calibration of the PLS-DA model shows that the best accuracy was obtained for two components (Figure 11A), which gathered information from all explanatory variables. The overall model precision was 0.84 on the training dataset and 0.8 on the test samples (Table 6).

A comparison of the model’s quality was shown on the plot representing the distribution of residuals (Figure 11A). Figure 11A shows that the PLS-DA model slightly outperforms the rest of them in a sense of residuals, but also has the highest variance.

The two classification models (PLR, RF) are black boxes, which means it is hard to trace the link between input variables and model outcomes [53]. Only regularized logistic regression (PR) is the kind of model in which the relation between explanatory and dependent variables is evident. To unify the assessment of variable importance, universal method was used [44]. All applied classification models (PLS-DA, PR, RF) confirm that the ϕ_0_ MeOH value is the most important predictor among all. The second most important is sMeOH (Figure 11B).

#### 3.7.3. Regression Model Comparisons

Similarly, to the classification approach, RF models were estimated for predictors from two types of feature selection. One could be noticed that feature algorithms were applied to the same data, but in the regression task, so the sets of variables chosen by them are different than in classification. Again, the RF model with initial RFE selection gives the best prediction. RMSE for the final model on the training dataset was equal to 145.72 but on test samples, an even better RMSE = 106.17.

The calibration of PLS regression shows that the best model was obtained for two components, where RMSE = 154.72 on the training dataset. Overall performance for test samples was better, because RMSE = 115.74 with R2 = 0.48, and MAE = 99.53.

Finally, estimation of Penalized Regression parameters was conducted and the best combination of hyperparameters was α = 10^−5^ and λ = 0.001. RMSE for the final model was 142.06 on the training dataset. RMSE on test samples was even lower at 112.20 with R^2^ = 0.44 and MAE = 92.75.

**Figure 12:**
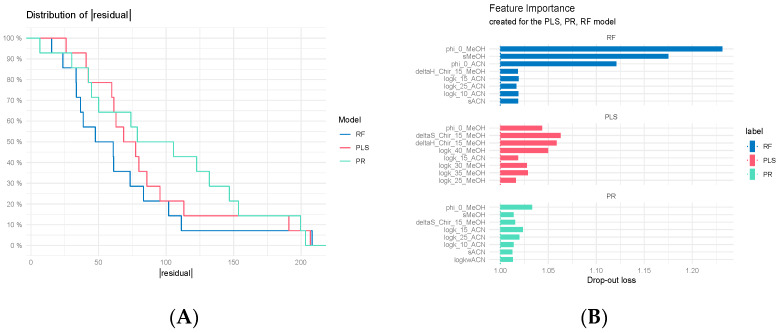
Distribution of residuals (**A**), variable importance for set of optimal RFE predictors (**B**) of three estimated models.

Based on the distribution of residuals, evaluated for each model (RF, PR, PLS), one could notice that RF is the best regression model because it outperforms the rest of the models in the sense of distribution of residuals (Figure 12A). Assessment of the variable importance in the regression models again pointed out that the most important variable is ϕ_0_ MeOH and the second most important is *S* MeOH (Figure 12B). Key variables were common to both types of models.

## 4. Conclusions

The drug, after administration, contacts many different biological membranes, i.e., macrophage cells, vascular endothelium, and complex absorption barriers. The interaction of the biomolecule with these membranes affects its further biodistribution in the body. To date, due to the complexity of the structure and function of various biological barriers, many simpler models have been proposed to study the in vitro interaction between bioactive compounds and biological membrane models. Due to the analogy between interactions occurring in the biological environment and chromatographic systems, the potential of the teicoplanin-modified HPLC column for predicting physicochemical parameters significant in ADMET processes has been studied in the current work.

In this paper, the chromatographic system with the teicoplanin modified column (Chirobiotic T) and organic-aqueous mobile phase was used to model the biological membrane. Although teicoplanin-bonded silica was first introduced as an enantioselective stationary phase, it has shown that it may also be useful to the analysis of achiral molecules.

Thermodynamic parameters obtained experimentally and calculated based on molecular modeling consistently confirm weak interactions of 1,2,4-triazolo-3-thiones with teicoplanin moiety. This is evidenced especially by small ΔG° values: theoretically estimated by the docking simulation in the range of −5.38 to −4.65 kJ/mol, experimentally on teicoplanin column and methanol/water eluent system in the range of −7.64 to −5.23 kJ/mol, and acetonitrile/water eluent system in the range of −6.02 to −4.16 kJ/mol. Despite this, the DFT-derived binding energies are systematically higher, and their correlation with experimental data is better (R^2^ = 0.8595 for MeOH, and 0.6814 for ACN). The weak interaction behavior of investigated analytes reflects the results of earlier pharmacological studies that showed poor permeability of these compounds through the blood-brain barrier despite the confirmed anticonvulsant activity.

The experimentally measured values of the so-called chromatographic lipophilicity parameter (log k_w_) are also significantly smaller than theoretically predicted, which further confirms the ability of teicoplanin to predict the behavior of the studied xenobiotics at the interface of biological barriers.

The usefulness of this model should be emphasized, especially with a methanol/water eluent system which is able to very precisely reflect the polarizability of molecules. This parameter appears to be important for the future selection of anticonvulsant drug candidates.

Teicoplanin generates both polar and hydrophobic chemical interactions, ensuring not only a partitioning phenomenon but also a binding pathway. Only on the basis of measurements of retention in the teicoplanin/methanol-water system, it is possible to initially anticipate both pharmacokinetic aspects of the behavior of the potential drug in vivo. It was proved by PLS-DA, that there is a potential to discriminate between active and inactive 1,2,4-triazole-3-thiones due to the chromatographically measured variables (φ_0_ MeOH, *S* MeOH). Summarizing, it can be concluded that the teicoplanin HPLC column possesses the potential to estimate 1,2,4-triazole-3-thiones’ antiepileptic properties.

## Figures and Tables

**Table 1:** Investigated compounds.

General Formula (1)		General Formula (2)	
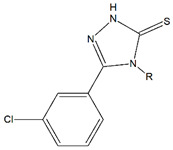		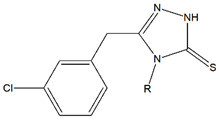	
No.	R	No.	R
TPR-2 AC log P = 3.93 * 310.3 mg/kg bw **	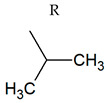	TPR-20 AC log P = 3.83 * (−) **	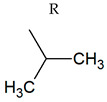
TPR-4 AC log P = 4.33 * 221.7 mg/kg bw **	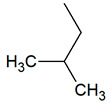	TPR-22 AC log P = 4.23 * 130.4 mg/kg bw **	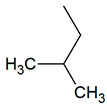
TPR-6 AC log P = 4.08 * 295.1 mg/kg bw **	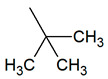	TPR-24 AC log P = 3.98 * (−) **	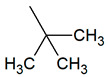
TPR-8 AC log P = 4.79 * (−) **	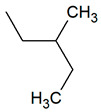	TPR-26 AC log P = 4.69 * (−) **	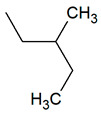
TPR-10 AC log P = 5.32 * (−) **	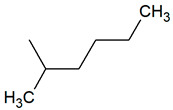	TPR-34 AC log P = 5.22 * (−) **	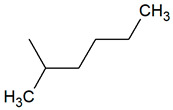
TPR-14 AC log P_s_ = 3.49 * 208.9 mg/kg bw **	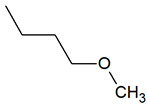	TPR-28 AC log P = 3.39 * 132.4 mg/kg bw **	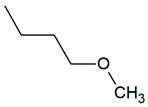
TPR-16 AC log P = 4.16 * 201.0 mg/kg bw **	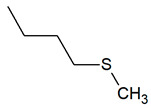	TPR-30 AC log P = 4.06 * 320.0 mg/kg bw **	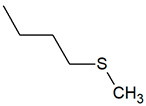

* AC log P—The logarithm of the theoretical partition coefficients of the examined solutes were calculated using ALOGPS 2.1–VCCLab, available free of charge on the following website: www.vcclab.org. ** The anticonvulsant activity of the compounds was expressed as ED50 values. Each ED50 reflects the dose of a compound required to protect 50% of the animals tested against maximal electroshock (MES)-induced seizures. The data were obtained from our previous paper [39]. The symbol: (−) means that the compound is inactive.

**Table 2:** Statistical estimates for the linear relationships (log *k* vs. the volume fraction of the organic modifier: methanol and acetonitrile) obtained on the Chirobiotic T column.

Symbol	MeOH						ACN					
	log *k*_w_	*S*	φ_0_	R^2^	F	s_e_	log *k*_w_	*S*	φ_0_	R^2^	F	s_e_
TPR 2	1.3574	−2.3728	0.5721	0.9902	404	0.0247	1.3794	−3.1428	0.4389	0.9784	182	0.0488
TPR 4	1.4772	−2.5712	0.5745	0.9925	526	0.0234	1.4850	−3.4647	0.4286	0.9796	192	0.0523
TPR 6	1.4200	−2.1986	0.6459	0.9804	200	0.0325	1.4859	−3.3270	0.4466	0.9843	250	0.0440
TPR 8	1.6778	−2.9497	0.5688	0.9937	630	0.0246	1.6600	−3.9492	0.4203	0.9863	289	0.0486
TPR 10	1.7921	−3.2712	0.5478	0.9956	913	0.0226	1.8393	−4.4832	0.4103	0.9856	273	0.0567
TPR 14	1.4238	−2.4081	0.5912	0.9920	2519	0.0126	1.4227	−3.3181	0.4288	0.9732	375	0.0419
TPR 16	1.7177	−2.8469	0.6034	0.9960	985	0.0190	1.6785	−3.9831	0.4214	0.9818	216	0.0567
TPR 20	1.4968	−2.6651	0.5616	0.9974	1518	0.0143	1.4711	−3.3363	0.4409	0.9765	166	0.0541
TPR 22	1.6746	−2.9338	0.5708	0.9983	2285	0.0128	1.5954	−3.6636	0.4355	0.9815	213	0.0526
TPR 24	1.6998	−3.0168	0.5635	0.9984	2519	0.0126	1.6752	−3.8815	0.4316	0.9895	375	0.0419
TPR 26	1.8619	−3.2460	0.5736	0.9979	1884	0.0156	1.8080	−4.3276	0.4178	0.9858	278	0.0543
TPR 28	1.5547	−2.6348	0.5901	0.9979	1884	0.0156	1.5212	−3.5573	0.4276	0.9858	278	0.0543
TPR 30	1.8527	−3.0514	0.6072	0.9972	1409	0.0170	1.7957	−4.2644	0.4211	0.9851	264	0.0549
TPR 34	2.0070	−3.6746	0.5462	0.9966	1161	0.0226	1.9553	−4.7297	0.4134	0.9915	466	0.0458
**Descriptive Statistics**												
Mean	1.644	−2.846	0.580	0.995	1345.6	0.019	1.627	−3.816	0.427	0.983	272.76	0.050
Median	1.676	−2.890	0.573	0.996	1285.3	0.018	1.628	−3.773	0.428	0.985	268.81	0.052
Standard deviation	0.188	0.388	0.025	0.005	755.2	0.006	0.169	0.476	0.010	0.005	81.19	0.005
Skewness	0.211	−0.316	1.195	−2.160	0.185	0.709	0.367	−0.422	0.183	−0.399	0.972	−0.557
Kurtosis	-0.873	−0.088	1.931	5.447	−1.240	0.022	−0.916	−0.968	0.714	−0.132	0.723	−1.171
Coefficient of variation	11.854	14.140	4.536	0.487	58.244	30.851	10.780	12.953	2.494	0.511	30.89	10.487

**Table 3:** Thermodynamic parameters obtained for investigated compounds by the use of the teicoplanin stationary phase and 15% organic-aqueous eluents with methanol or acetonitrile.

MeOH							ACN					
No TPR	∆H° kJ/mol	∆S°^^^ kJ/mol*K	∆G° kJ/mol	R^2^	F	Se	∆H° kJ/mol	∆S° kJ/mol*K	∆G° kJ/mol	R^2^	F	Se
2	−20.6090	−0.0521	−5.2303	0.9979	2882	0.017	−8.3367	−0.0142	−4.1676	0.8175	27	0.072
4	−20.5715	−0.0502	−5.7669	0.9999	50484	0.004	−7.7317	−0.0111	−4.4872	0.8404	32	0.062
6	−16.4897	−0.0355	−5.9824	0.9824	334	0.040	−4.1104	0.0022	−4.7408	0.3960	4	0.093
8	−23.5131	−0.0569	−6.7458	0.9999	46120	0.005	−8.8404	−0.0129	−5.0677	0.8828	45	0.059
10	−22.4397	−0.0524	−6.9865	0.9996	16937	0.008	−10.0121	−0.0151	−5.5932	0.8824	45	0.067
14	−23.4591	−0.0598	−5.8228	0.9995	21550	0.007	−9.8373	−0.0194	−4.1642	0.9670	131	0.045
16	−27.5706	−0.0693	−7.1638	0.9995	12907	0.011	−13.4231	−0.0281	−5.1833	0.9629	156	0.048
20	−21.4193	−0.0534	−5.6540	0.9997	19402	0.007	−10.2859	−0.0198	−4.4765	0.9668	175	0.035
22	−22.7078	−0.0552	−6.4215	0.9999	44485	0.005	−10.2560	−0.0183	−4.8815	0.9624	154	0.037
24	−22.0695	−0.0528	−6.4815	0.9997	21550	0.007	−11.9776	−0.0231	−5.1955	0.9562	131	0.047
26	−24.5931	−0.0586	−7.3147	0.9994	10895	0.011	−11.0007	−0.0189	−5.4615	0.9597	143	0.041
28	−25.2271	−0.0643	−6.2751	0.9995	12046	0.010	−11.2507	−0.0232	−4.4612	0.9779	266	0.031
30	−30.1099	−0.0763	−7.6480	0.9996	13413	0.012	−14.9553	−0.0323	−5.4933	0.9814	317	0.038
34	−25.7792	−0.0615	−7.6401	0.9999	48330	0.005	−13.1747	−0.0244	−6.0226	0.9618	151	0.048

**Table 4:** Cross-correlations of ΔG° and log *k*_w_ estimated in methanol and acetonitrile eluent system with the theoretical descriptors.*

Log P_hyper_	HE	Polarizability	SA	Vol	µ	HF	HOMO	LUMO	ALOGPs	AC logP	ALOG P	MLOG P	XLOG P2	XLOG P3	SVM BBB	AdaBoost BBB
**Cross-correlation with ΔG° estimated in methanol/water eluent system**																
0.005	0.000	0.921	0.037	0.670	0.043	0.001	0.406	0.011	0.143	0.361	0.282	0.385	0.302	0.339	0.397	0.119
**Cross-correlation with ΔG° estimated in acetonitrile/water eluent system**																
0.113	0.105	0.684	0.006	0.333	0.000	0.139	0.167	0.129	0.394	0.626	0.584	0.687	0.685	0.618	0.215	0.007
**Cross-correlation with log *k*_w_ estimated in methanol/water eluent system**																
0.036	0.015	0.886	0.007	0.607	0.015	0.003	0.420	0.003	0.245	0.427	0.377	0.458	0.434	0.417	0.328	0.043
**Cross-correlation with log *k*_w_ estimated in acetonitrile/water eluent system**																
0.110	0.094	0.689	0.016	0.335	0.002	0.068	0.202	0.065	0.406	0.642	0.598	0.681	0.687	0.630	0.234	0.009

* The theoretical descriptors were calculated with software available free of charge available at https://preadmet.bmdrc.kr/adme/, https://www.cbligand.org/BBB/predictor.php.

**Table 5:** The docking and DFT (density functional theory) results obtained with respect to the studied ligands. The docking calculations were averaged over four teicoplanin structures available in the PDB (Protein Data Bank) records; the corresponding standard deviations are given.

Ligand	Binding Energy, Docking (kJ/mol)	Binding Energy, DFT (kJ/mol)	Ligand	Binding Energy, Docking (kJ/mol)	Binding Energy, DFT (kJ/mol)
TRP-2	−5.15 ± 0.53	−10.22	TRP-20	−5.05 ± 0.29	−11.47
TRP-4	−4.95 ± 0.33	−10.88	TRP-22	−5.30 ± 0.49	−13.57
TRP-6	−4.98 ± 0.11	−12.62	TRP-24	−5.38 ± 0.29	−12.10
TRP-8	−4.90 ± 0.41	−13.47	TRP-26	−5.05 ± 0.42	−13.96
TPR-10	−4.68 ± 0.23	−14.93	TPR-28	−5.05 ± 0.38	−12.56
TRP-14	−4.88 ± 0.31	−12.98	TRP-30	−4.80 ± 0.37	−16.46
TRP-16	−4.65 ± 0.21	−14.59	TRP-34	−5.30 ± 0.44	−15.96

**Table 6:** Metrics for classification of PR, RF and PLS-DA on test data.

Prediction: Active/Nonactive	PR—Regularized Logistic Regression	RF—Random Forest	PLS-DA
Accuracy	0.8	0.8	0.8
95% CI	(0.2836, 0.9949)	(0.2836, 0.9949)	(0.2836, 0.9949)
No Information Rate	0.6	0.6	0.6
*p*-Value (Acc > NIR)	0.337	0.337	0.337
Kappa	0.5455	0.5455	0.5455
McNemar’s Test *p*-Value	1.000	1.000	1.000
Sensitivity	1.00	1.00	1.00
Specificity	0.50	0.50	0.50
Pos Pred Value	0.75	0.75	0.75
Neg Pred Value	1.00	1.00	1.00
Prevalence	0.60	0.60	0.60
Detection Rate	0.60	0.60	0.60
Detection Prevalence	0.80	0.80	0.80
Balanced Accuracy	0.75	0.75	0.75
Positive class	active	active	active
